# A rotary transformer cross-subject model for continuous estimation of finger joints kinematics and a transfer learning approach for new subjects

**DOI:** 10.3389/fnins.2024.1306050

**Published:** 2024-03-20

**Authors:** Chuang Lin, Zheng He

**Affiliations:** School of Information Science and Technology, Dalian Maritime University, Dalian, China

**Keywords:** sEMG, rotary transformer (RoFormer), cross-subject model, transfer learning, finger joint angles estimation

## Abstract

**Introduction:**

Surface Electromyographic (sEMG) signals are widely utilized for estimating finger kinematics continuously in human-machine interfaces (HMI), and deep learning approaches are crucial in constructing the models. At present, most models are extracted on specific subjects and do not have cross-subject generalizability. Considering the erratic nature of sEMG signals, a model trained on a specific subject cannot be directly applied to other subjects. Therefore, in this study, we proposed a cross-subject model based on the Rotary Transformer (RoFormer) to extract features of multiple subjects for continuous estimation kinematics and extend it to new subjects by adversarial transfer learning (ATL) approach.

**Methods:**

We utilized the new subject’s training data and an ATL approach to calibrate the cross-subject model. To improve the performance of the classic transformer network, we compare the impact of different position embeddings on model performance, including learnable absolute position embedding, Sinusoidal absolute position embedding, and Rotary Position Embedding (RoPE), and eventually selected RoPE. We conducted experiments on 10 randomly selected subjects from the NinaproDB2 dataset, using Pearson correlation coefficient (CC), normalized root mean square error (NRMSE), and coefficient of determination (R2) as performance metrics.

**Results:**

The proposed model was compared with four other models including LSTM, TCN, Transformer, and CNN-Attention. The results demonstrated that both in cross-subject and subject-specific cases the performance of RoFormer was significantly better than the other four models. Additionally, the ATL approach improves the generalization performance of the cross-subject model better than the fine-tuning (FT) transfer learning approach.

**Discussion:**

The findings indicate that the proposed RoFormer-based method with an ATL approach has the potential for practical applications in robot hand control and other HMI settings. The model’s superior performance suggests its suitability for continuous estimation of finger kinematics across different subjects, addressing the limitations of subject-specific models.

## 1 Introduction

Surface electromyography (sEMG) is obtained by recording the electrical activity of muscles through the skin and can reflect the electrical activity of muscle fibers when they contract. sEMG has been widely used in intelligent prostheses, external skeletal robotic arms, space remote operations, industrial robot control, etc. It can be integrated with deep learning techniques for regression-based kinematics estimation to intuitively comprehend human intents.

Recent advancements in deep learning (DL) technology, particularly attention-based Transformer models, have garnered considerable public interest, shifting the artificial intelligence paradigm from classic feature engineering to feature learning. For instance, a new convolutional visual transformer (CviT) with stacked set learning was proposed by Shen et al. (2022). and has tremendous potential for merging the sequential and spatial aspects of sEMG signals with the parallel training below. Burrello et al. (2022) proposed using Bioformers, a supersmall attention-based neural network architecture, to solve the problems of big resource consumption and difficult improvement of accuracy in sEMG signal gesture recognition tasks. Montazerin et al. (2022) proposed a gesture recognition model called ViT-HGR, which is the first to introduce visual transformer architecture into high-density sEMG signal gesture recognition tasks, leveraging its parallel computing advantages to overcome the long training time and memory limitations of deep learning models. Liu et al. (2023) proposed a CNN-Transformer hybrid network for high-accuracy dynamic gesture recognition. It adopts continuous wavelet transform to obtain time-frequency representations of sEMG signals, uses attention mechanisms to combine local and global features, and achieves gesture recognition through multi-branch and multi-scale fusion to improve recognition accuracy and efficiency. However, the majority of these outcomes were produced in straightforward, static laboratory settings. External influences, such as muscle fatigue, electrode displacement, and the impact of arm posture, can easily affect the features of sEMG (Hill et al., 2016; Kusche and Ryschka, 2019). Chen et al. (2022) proposed an extended spatial transformer convolutional neural network (EST-CNN) model, which automatically learns electrode displacement relationships through feature enhancement preprocessing and spatial transformation layers, further micro-adjusts rotation angles through tuning layers, can simultaneously achieve gesture recognition and autonomous motion calibration, and effectively improve recognition accuracy under electrode movement. Especially, sEMG signals have user-specific characteristics, resulting in large differences in amplitude and frequency between subjects even when collected from the same position with the same movement (De Luca, 2002). Recently, a BERT model has been proposed for estimating hand kinematics from sEMG signals, achieving SOTA performance in cross-subject cases (Lin et al., 2022). However, the test data is not independent of the model’s training set.

Despite the fact that certain research implies that features in different subjects may have similar distributions (Xu et al., 2018), subject variability still leads to a dramatic drop in the previously trained model’s estimation performance (Xiong et al., 2021). Transfer learning approaches (Pan and Yang, 2010) can adjust the model so that it can be applied to other subjects. Fine-tuning (FT) is a popular deep transfer learning method although it might overfit when the target domain has insufficient labeled data. In addition to FT, domain adaptation (DA) is also a prominent TL approach, which enhances the target prediction function by minimizing the difference between the source and target domain feature distributions, requiring fewer target data while maintaining consistent performance compared to fine-tuning. Ketykó et al. (2019) proposed a domain adaptation method that enhances the accuracy of recognition between sparse and high-density sEMG signals through the approximating domain transfer, addressing inter-session and inter-subject differences. Du et al. (2017) proposed a deep-learning based domain adaptation method for intra-session and inter-session gesture recognition tasks, addressing the limitation of conventional methods relying on a single session. Shi et al. (2022) proposed a multi-task dual-stream supervised domain adaptation network MDSDA based on CNN for long-term multi-subject gesture recognition using sEMG signals. Experimental results show that MDSDA outperforms conventional CNN and fine-tuning in a long-term multi-subject environment, and static and dynamic gestures have separability, which helps reduce signal collection burden.

These recent studies on domain adaptation have taken an important step toward building cross-subject transfer learning models, but they are limited to gesture recognition and cannot be utilized to continuously estimate finger joint angles. Considering the complex anatomical structure and kinematic characteristics of the hand, it is more challenging to build a cross-domain universal model for finger kinematics estimation. Although the BERT-based cross-subject method (Lin et al., 2022) can be applied to several subjects simultaneously, it relies on new subject data during the model training phase. As a result, we present a new cross-subject model based on adversarial transfer learning (ATL) in this study to estimate the finger joint angles of new subjects independently of model training. First, a RoFormer model was established using sEMG and finger kinematics data from several subjects. The RoFormer model is then calibrated utilizing ATL and training data from new subjects. The suggested RoFormer model’s generalization performance to new individuals is verified on the NinaproDB2 dataset. In summary, the main contributions of this paper are:

- The RoFormer-based cross-subject model for continuous hand movement regression was proposed for the first time, and the experimental results show that our method reaches state-of-the-art performance both in cross-subject and subject-specific cases.
- An ATL approach was proposed to transfer knowledge from several subjects to a new subject in an adversarial learning manner, significantly improving the generalization ability of cross-subject models.

The outcomes obtained in this work show great potential in practical applications of robot hand control and will greatly promote the application of other HMI settings.

## 2 Related work

### 2.1 Long short-term memory network (LSTM)

When processing long sequence data, Recurrent neural network (RNN) models are prone to the problems of vanishing and exploding gradients, which can cause the model to fail to effectively capture long-term dependencies. LSTM (Hochreiter and Schmidhuber, 1997) is a form of RNN that aims to address the long-term dependency issue in typical RNN models through a combination of memory and forget gates.

In this study, the LSTM architecture was used as a comparison, with 4 interaction layers in each cell. We utilized a five-layer stacked LSTM to boost the model’s depth to improve its fitting ability. Each layer had a hidden dimension of 32.

### 2.2 Temporal convolutional network (TCN)

Due to the limitations of the convolutional kernel size, traditional convolutional neural networks are incapable of capturing long-term dependencies, making them unsuitable for modeling time-series data. To address this issue, TCN (Bai et al., 2018) was proposed. The three basic components of TCN are residual convolution, dilated convolution, and causally connected convolution. The one-way structure of causally connected convolution prevents it from seeing future data. TCN utilizes dilated convolution (Yu and Koltun, 2015) to increase the receptive field and decrease the linearly stacked CNN layers to capture longer relationships. TCN adopts a 1D fully convolutional network (FCN) (Long et al., 2015) structure with hidden layers that are identical in length to the input layer.

In this study, we choose to use a total of 6 layers, comprising 1 completely connected layer and 5 dilated causally connected convolution layers with residual blocks. The first five layers’ convolutional channels are set up as 32, 64, 32, and 10, respectively. Stride and kernel sizes are set to 3 and 1, respectively.

### 2.3 Transformer network

The Transformer neural network proposed by Vaswani et al. (2017) has been widely adopted in natural language processing (Devlin et al., 2018) and speech recognition (Krishna et al., 2019). Transformer utilizes a combination of encoders and decoders to handle input and output sequences. In this study, only the encoder part is employed to estimate hand motions from sEMG signals. The Transformer Encoder is made up of several stacked blocks, each with two sub-layers: the Multi-Head Self-Attention Mechanism (MSA) and the Feed-Forward Neural Network (FFNN).

The self-attention (SA) mechanism computes the relevance of each position in the input sequence to all other positions, enabling the model to effectively capture long-range dependencies. The MSA mechanism enhances this capability by dividing the input information into multiple “heads,” which are then individually processed using SA. This enables the model to capture diverse features of the input information in separate representation spaces. The FFNN is also known as the Fully Connected (FC) Layer.

Furthermore, the Transformer model incorporates positional encoding to retain the positional information of each element in the sequence. Layer normalization and residual connections are applied in each attention and feed-forward layer before a final FC layer maps features to 10 joint angles. In this study, we stacked two encoder layers, each MSA module consists of five SA modules.

### 2.4 CNN-attention network

There are two main components of the CNN-Attention model: a multi-scale convolution module and a multi-head self-attention (MSA) module (Geng et al., 2022). The multi-scale convolution module is made up of three parallel routes with convolution kernel sizes of three, five, and seven. To ensure that the sequence lengths of the input and output are equal, the convolution utilizes the proper padding. The average pooling layer receives the outcome of the first multi-scale convolution module to shorten the feature sequence and broaden the convolution’s receptive field. Then, an MSA architecture is utilized to estimate joint angles. In the present research, we stacked three MSA modules, each MSA module is made up of three single-headed attention.

## 3 A transfer learning model based on RoFormer

### 3.1 RoFormer

While the previous models discussed have applicability in our field, some limitations remain. LSTM relies on prior data for training because of its RNN structure, it is challenging to accomplish hardware parallel acceleration, which leads to subpar real-time performance. TCN relies on CNN structure rather than RNN structure, resulting in unstable and fluctuating estimates. We also compare the recently proposed models CNN-Attention and Transformer, both of which are based on attention mechanisms. The model could be utilized to process extremely lengthy time sequences thanks to the design of parallel computing structure and attention mechanism. This is one of its unique advantages over RNN and CNN. Another thing they have in common is the use of absolute position encoding. Absolute position embedding has the advantages of being simple to implement and having a rapid calculation time, but its performance is not as good as the network using relative position coding.

Therefore, we adopt a rotary transformer (RoFormer) neural network, which uses Rotary Position Embedding (RoPE) instead of absolute position embedding in traditional transformers to improve the accuracy of continuous estimation of finger motions. The basic idea of RoPE is to enable the model to focus on relative positions through absolute position encoding. This model is introduced below.

#### 3.1.1 Rotary position embedding (RoPE)

Su et al. (2021) pioneered the rotary position embedding (RoPE) method. Rather than directly adding sinusoidal embedding as in the original Transformer, they multiply the keys and queries of each attention layer by sinusoidal embedding.

In contrast to sinusoidal or learnable positional embedding, RoPE injects position information at each layer rather than simply the first. Furthermore, the values vector of the self-attention (SA) layer does not receive any positional information. Because the output of an SA layer is a linearly transformed, weighted sum of the input value vectors, the outputs of each SA layer include no explicit position information because position information is not inserted into the values. We adopted RoPE in our model.

#### 3.1.2 Model structure

**Figure 1:**
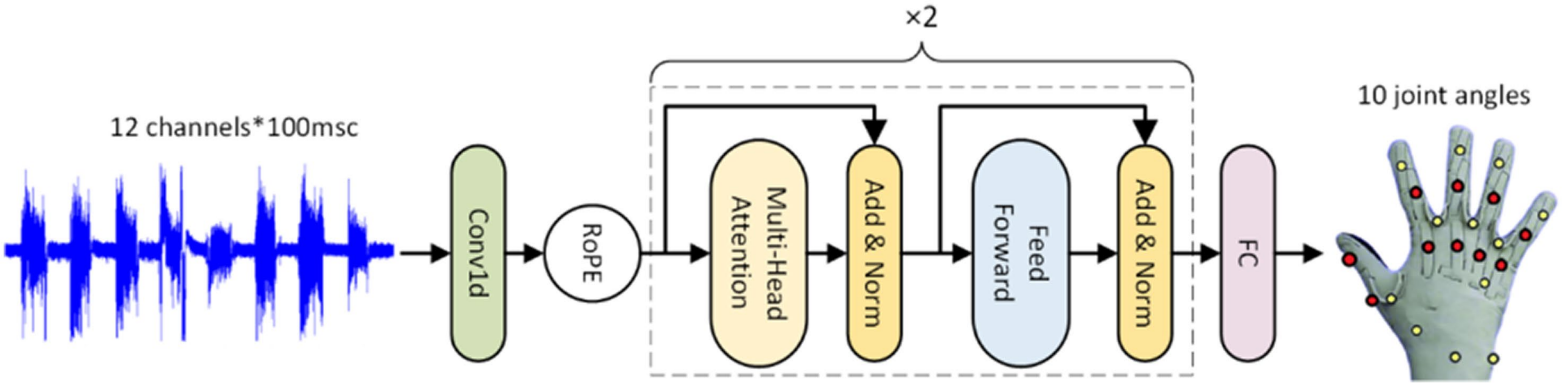
The construction of the rotary transformer(RoFormer) module. RoPE represents rotary position embedding. The sEMG’s temporal information can be captured by the model thanks to RoPE.

To decrease the number of model parameters and accelerate convergence, we add a 1D convolutional neural network layer before RoFormer. In Figure 1, the procedure is depicted and explained.

The multi-head self-attention (MSA) and multi-layer perceptron network (MLP) modules make up the Transformer encoder. The MSA module calculates the correlation between each position element of the input sequence, and the MLP module performs deep transformation on each position element to obtain higher-level representation capabilities.

To provide a clearer description of the model, we represent the 1-D input series as: $X=\left[x_{0},\;x_{1},\cdots ,\;x_{t}\right]$ and is then fed to a linear projection layer (E) to get the $X^{p}=\left[x_{0}^{p}E,\;x_{1}^{p}E,\cdots ,\;x_{t}^{p}E\right]$ . The output of each layer of the encoder is defined as $Z_{i}\left(1\leq i\leq L\right)$ , L is the number of encoder layers. We execute rotary position embedding (RoPE) on input before feeding it into these encoder layers, designating as $Z_{0}$ (Eq. 1.1):

(1.1) $$Z_{0}=\mathrm{RoPE}\left(X^{p}\right)$$

where represents the output after $X^{p}$ input to RoPE. The encoder uses layer normalization and residual skip connections to overcome the degradation issue. With this approach, each encoder layer can be characterized as (Eq. 1.2):

(1.2) $$\begin{array}{c} Z_{l}^{\prime }=MSA\left(\mathrm{LayerNorm}\left(Z_{l-1}\right)\right)+Z_{l-1}\\ Z_{l}=MLP\left(\mathrm{LayerNorm}\left(Z_{l}^{\prime }\right)\right)+Z_{l}^{\prime } \end{array}$$

where $\left(1\leq i\leq L\right)\text{,}$ finally, A linear layer is used to retrieve outcomes from $Z_{L}$ (Eq. 1.3):

(1.3) $$Z=\mathrm{Linear}\left(Z_{L}\right)$$

Following that, we give an introduction to the MSA and MLP modules. The self-attention (SA) mechanism can be thought of as a method for identifying relational dependencies among various sample points within the input $Z_{i}$ , which is accomplished via the queries matrix (*Q*), the key matrix (*K*), and the values matrix (*V*). By using a linear transformation, they are calculated (Eq. 1.4):

(1.4) $$\left[Q,K,V\right]=ZW_{QKV}\text{,}$$

The weight matrix is learnable, and after scaling *Q* and *K*, they are then used to compute the weights of *V*. The final result (Eq. 1.5) is obtained by taking the weighted sum of all the values of *V*:

(1.5) $$SA\left(Z\right)=\mathrm{softmax}\left(\frac{QK^{T}}{\sqrt{d_{h}}}\right)V\text{,}$$

The MSA block contains H separate attention heads that process inputs in parallel. Each head has its own learnable parameters and performs self-attention independently. The outputs from all H heads are then concatenated and projected to produce the final MSA result. This is an illustration of the MSA (Eq. 1.6):

(1.6) $$MSA\left(Z\right)=\left[SA_{1}\left(Z\right);\;SA_{2}\left(Z\right);\ldots ;SA_{h}\left(Z\right)\right]W^{MSA}$$

$W^{MSA}$ represents a learnable weight matrix in each encoder layer. In addition, the MLP module is made up of two linear layers, with the Mish activation function (Mish, 2019) coming after the first layer to boost accuracy and convergence speed.

Due to the powerful ability of attention mechanisms and residual skip connections to extract features from small-scale sequential data, RoFormer is also feasible in multiple subjects. RoFormer can receive both future and past signals simultaneously, allowing it to extract features from the entire sequence rather than just the past signals. This makes RoFormer superior to classical TCN and RNN models in cross-subject situations. The experiments part depicts the performance of RoFormer on NinaproDB2.

### 3.2 *μ*-law normalization

The sEMG signals of various subjects lie in distinct ranges with different distributions, in which Z-Score normalization may disturb their own features when several subjects are analyzed together. Furthermore, a significant amount of important information on sEMG in the temporal domain lies near zero (Rahimian et al., 2020) and cannot be identified by linear method.

The RMS characteristic of sEMG is amplified using a nonlinear logarithmic scaling method known as *μ*-law normalization (Recommendation ITU-T, 1988), taking into account the current low amplitudes of multiple sEMG channels. The *μ*-law normalization calculation formula is (Eq. 1.7):

(1.7) $$F\left(x_{t}\right)=\mathrm{sign}\left(x_{t}\right)\frac{\ln\left(1+\mu |x_{t}|\right)}{\ln\left(1+\mu \right)}$$

where t is time, Each channel’s sEMG signal is represented by $x_{t}$ , and *μ* is the scaling parameter for the signal. In our experiment, *μ* is set to $2^{20}$ , which has been searched from 256 to $2^{20}$ in the simulations. It has been proven that normalizing sEMG signals using the *μ*-law method can improve the network’s discrimination capability (Rahimian et al., 2021). The enhancement of regression model performance by the *μ*-law method is illustrated in the experiments chapter, especially in the cross-subject case.

### 3.3 Adversarial transfer learning (ATL) approach

**Figure 2:**
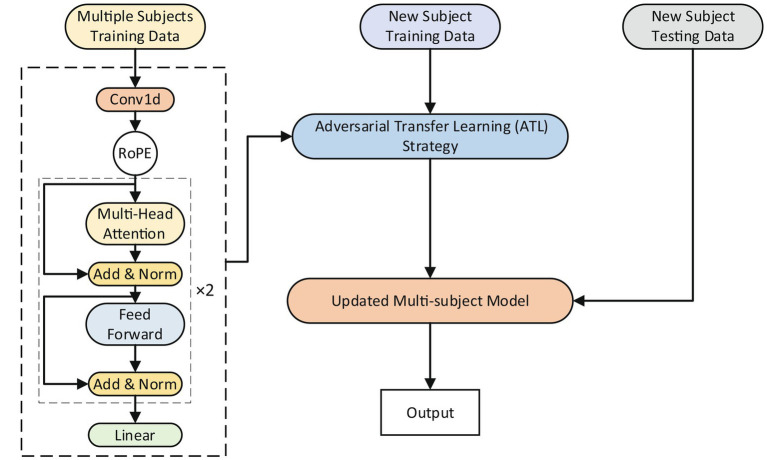
The overall framework of the cross-subject model and the adversarial transfer learning (ATL) approach.

**Figure 3:**
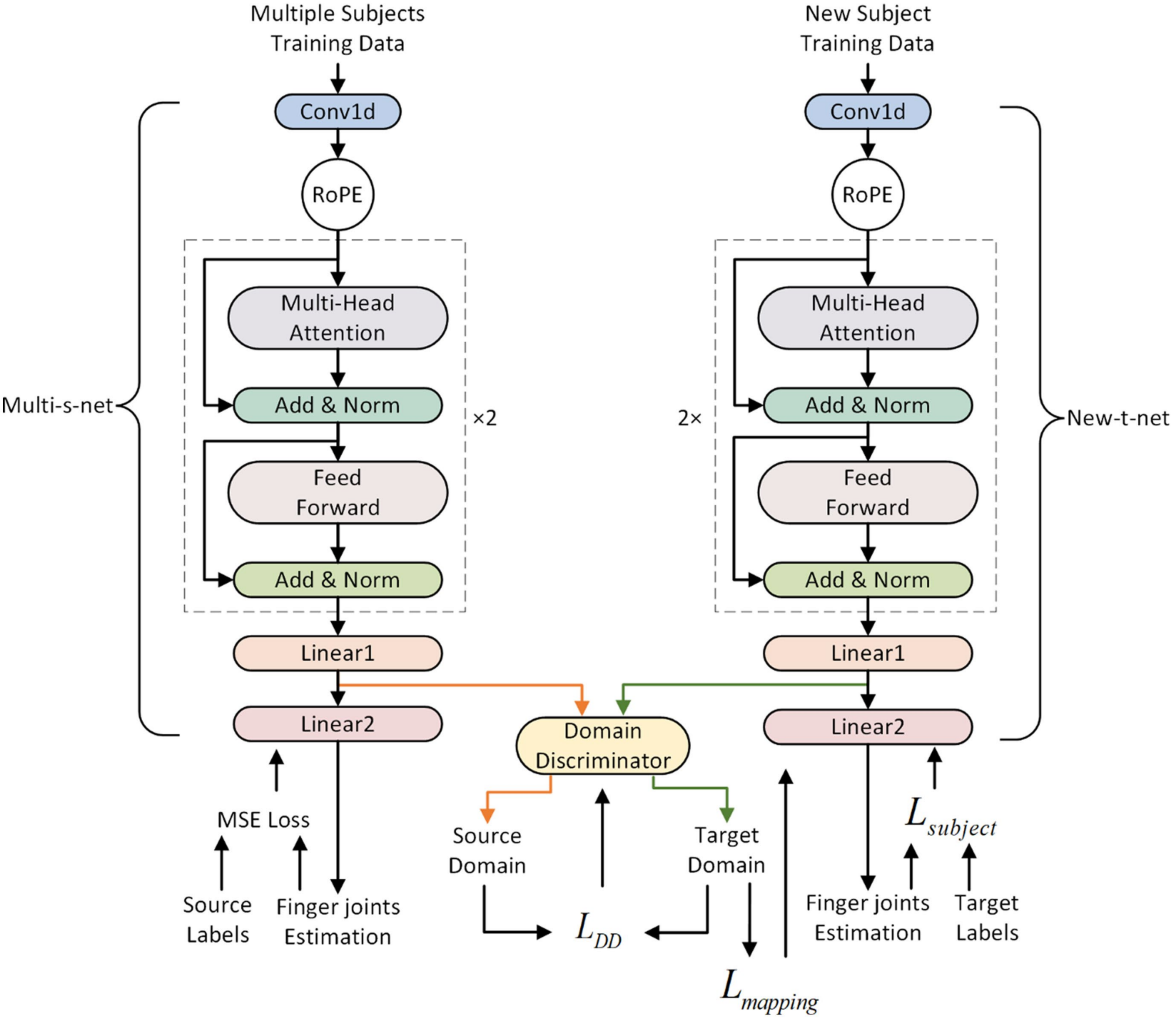
The adversarial transfer learning (ATL) approach. The DD is made up of MLP. The Source Labels refer to the finger joint angles labels from multiple subjects, while the Target Labels refer to the finger joint angles labels from a new subject.

The proposed model’s overall framework is shown in Figure 2. The RoFormer network and adversarial transfer learning (ATL) approach make up its two key components. The RoFormer network can act as a model for multiple subjects or as a model applied to an individual subject. The RoFormer cross-subject model was calibrated using the ATL approach, which allows the mapping of features from several subjects to new individuals. The ATL approach is briefly explained as follows:

The effectiveness of transfer learning approaches in transferring prior knowledge from the source to the target domains has been demonstrated (Du et al., 2017; Ketykó et al., 2019; Shi et al., 2022). The present study proposes an ATL approach to transfer knowledge gained from multiple subjects to new individuals in an adversarial manner (Goodfellow et al., 2020) while calibrating the parameters of the cross-subject model. The calibration process utilizing the ATL approach is depicted in Figure 3. A multi-subject source domain network (Multi-s-net) is used to extract source domain (data from multiple subjects) features. A new subject target domain network (New-t-net) is used to extract target domain (data from new individuals) features. A domain discriminator (DD) works toward minimizing the disparity between the feature distributions of the source and target domains. The DD consists of Multilayer Perceptron (MLP). Multi-s-net, New-t-net, and DD comprise the ATL transfer learning approach, facilitating both domain adaptation and multi-subject model calibration.

Before transfer learning, the Multi-s-net is trained using supervised mean square loss on data from multiple subjects. Multi-s-net and New-t-net share the same network architecture, with New-t-net initialized using the trained weights from Multi-s-net. During the process of ATL, the weights of the Multi-s-net remain fixed, while the weights of the New-t-net and DD are tuned to reduce the feature disparity between the source and target domains. The DD works to minimize domain discrepancy by attempting to differentiate between the two domains. Successful domain confusion by DD implies it can no longer distinguish the domains, indicating the minimum feature distance has been attained and knowledge transfer completed.

Drawing on the principles of adversarial learning (Goodfellow et al., 2020), the end-to-end adversarial transfer learning process is realized through formulae (1.8)–(1.11). Traditional transfer learning generally focuses on aligning marginal distributions across domains while neglecting feature consistency. Therefore, the current study optimizes DD and New-t-net using the loss function $L_{\mathrm{total}}$ to jointly account for:

(1.8) $$L_{\mathrm{total}}=L_{DD}+L_{\mathrm{mapping}}+L_{\mathrm{subject}}$$

$L_{\mathrm{total}}$ consists of $L_{DD}$ , $L_{\mathrm{mapping}}$ and $L_{\mathrm{subject}}$ . Where $L_{DD}$ represents the loss function of DD, which is utilized to measure the domain discrepancy between the feature spaces of New-t-net and Multi-s-net.

(1.9) $$L_{DD}=-\log\left(DD\left(F_{s}\right)\right)-\log\left(1-DD\left(F_{t}\right)\right)$$

Where $F_{s}$ refers to features from multiple subjects, $F_{t}$ refers to features from the new subject.

The sum of $L_{\mathrm{mapping}}$ and $L_{\mathrm{subject}}$ represents the loss function of New-t-net. Where $L_{\mathrm{mapping}}$ represents the degree of feature mapping from Multi-s-net to New-t-net. It can be described as:

(1.10) $$L_{\mathrm{mapping}}=-\log\left(DD\left(F_{t}\right)\right)$$

To make New-t-net more sensitive to the new subject’s features during ATL, $L_{\mathrm{subject}}$ reweights the loss function.

(1.11) $$L_{\mathrm{subject}}=w\overset{N}{\underset{1}{\sum }}{\left(\mathrm{Nnet}\left(x\right)-\hat{x}\right)}^{2}$$

Where $\mathrm{Nnet}\left(x\right)$ refers to the output of New-t-net, $\hat{x}$ represents the labels of finger joint angles, *N* represents finger joint numbers which equals 10 in this study and w is a weighting coefficient.

The DD consists of Multilayer Perceptron (MLP). MLP is a neural network model with strong adaptive and self-learning capabilities. It should be noted that in this study, the features output by Multi-s-net (denoted with orange lines with arrows) and the features output by New-t-net (denoted with green lines with arrows) are input into DD respectively, as illustrated in Figure 3. These features are the output of the penultimate fully connected layer in the RoFormer network.

In this research, all of the models are constructed based on the Pytorch 2.0.1 framework and trained on an NVIDIA RTX 3060 GPU. For training subject-specific and cross-subject models, the batch size is equal to 64, and the number of epochs is 400. The cross-subject model learning rate is initially fixed at 1e-4, and the subject-specific model learning rate is initially set at 3e-4, both of them are halved after 200 epochs. While calibrating the cross-subject model with new users, the batch size is 64, the calibration rate is 1e-3, and the calibration period consists of 50 epochs.

## 4 Experiments

### 4.1 Dataset

**Figure 4:**
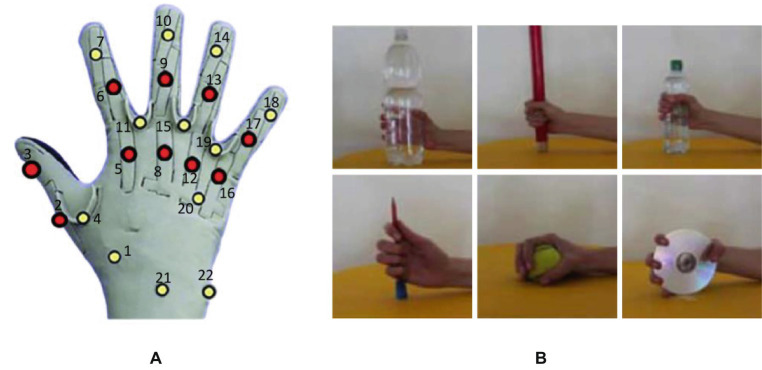
Dataset Description. **(A)** Cyber Glove II with 22 channels, and we picked up 10 finger joint angles, which are indicated by red dots. **(B)** Six grasping movements.

The Ninapro database (Atzori et al., 2015) is the largest, publicly accessible hand sEMG database in the field. To facilitate evaluation, the RoFormer model is evaluated using the Ninapro database. We utilize the second Ninapro database (also known as DB2), which contains sEMG signal recordings of 40 healthy subjects in 50 postures, such as wrist, hand, grasping, and functional movement patterns. The raw sEMG signals were collected from 12 electrodes using the Delsys Trigno wireless system, with a sampling frequency of 2 kHz. Simultaneously, the 22-sensor CyberGlove II data glove measures the hand kinematics at a sample frequency of 20 Hz before resampling to 2 kHz. Here, we use 12-channel sEMG signals to estimate the finger angles of hand movements in Figure 4A. Moreover, we picked up six grasping actions for different objects are shown in Figure 4B for each participant.

1. Subject selection: In this study, we selected 10 typical subjects from Ninapro DB2 for experiments. These subjects covered as much as possible all the subjects in the database in terms of height, weight, gender, age, and handedness. Range of characteristics of the examinee. For each subject, we selected 6 representative grasping actions based on the shape and diameter of the object (Figure 4B). The main shapes include cylinders, spheres, and flat objects, and the diameters cover objects of large, medium, and small sizes.
2. Data preprocessing: To reduce the impact of noise during data collection, we extract the root mean square (RMS) feature of sEMG signals using a 100 ms sliding window and a step size of 0.5 ms. These features are then normalized using the μ -law method to scale the amplitude range of the sensor output.

To perform training and testing experiments on our proposed RoFormer network, for all subject-specific and cross-subject cases as well as model calibration for each new user, each movement (which included six trials) was divided into training and testing datasets. Four randomly chosen trials out of a total of six trials make up the training dataset; the other two trials are the testing dataset.

### 4.2 Performance metrics

1. Pearson correlation coefficient (CC) is a widely adopted metric for quantifying the linear correlation between two variables. It assesses the extent of similarity between the expected motion and the estimated motion. CC has a value between −1 and 1. A higher CC value indicates a stronger correlation, indicating a more accurate estimation of the motion.
2. Normalized Root Mean Square Error (NRMSE) is a metric for measuring prediction accuracy. It scales RMSE values for meaningful comparisons across angles. Smaller NRMSE indicates better estimation. With NRMSE, the estimation errors are scaled to [0, 1] uniformly for all angles.
3. Coefficient of determination (R2) is a measurement of a regression model’s ability to accurately predict the data. The value range of R2 is 0–1. It shows the percentage of the dependent variable’s variance that can be explained by the independent variables. The greater the R2 value represents the better estimation performance. Hence, we utilized R2 to calculate the variance between each joint angle’s measured and estimated values.

### 4.3 Experimental results

#### 4.3.1 The effect of rotary position embedding

**Figure 5:**
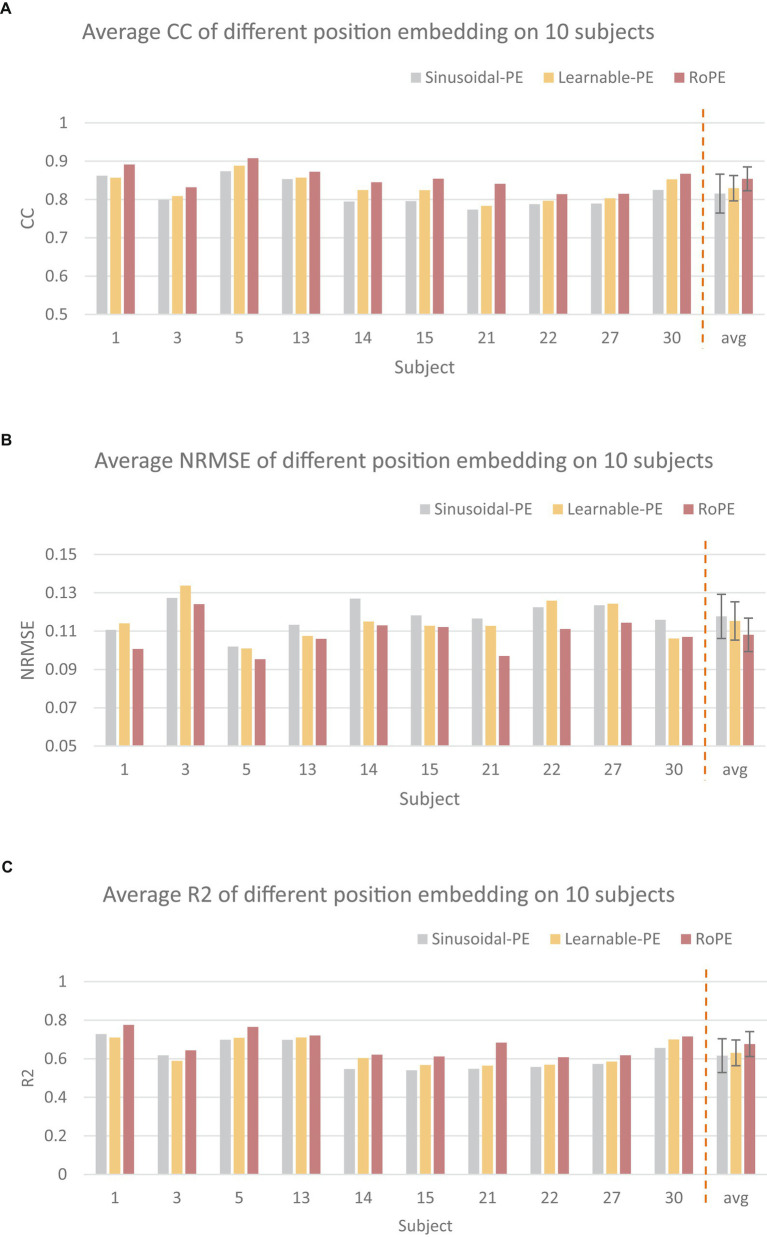
Comparison of different position embedding (PE). The CC **(A)**, NRMSE **(B)**, and R2 **(C)** were utilized as the performance metrics, respectively.

Figure 5 illustrates the performance of the RoFormer network applied with RoPE to continuously estimate the finger joint angle of each specific subject, and compares it with the transformer network applying absolute position embedding, including learnable position embedding (Learnable PE) and sinusoidal position embedding (Sinusoidal PE). In keeping with our proposed RoFormer model, the original input is fed into the transformer model after passing through a 1D convolutional neural network (conv1d) layer. The results show that the model applied with RoPE has an average CC of 0.8539 ± 0.029 an average NRMSE of 0.1080 ± 0.0087 and an average R2 of 0.6763 ± 0.064, which is better than the other two embedding. Compared to learnable position encoding, these values are 2.4% higher 0.0072 lower, and 4.6% higher respectively; compared to sinusoidal position encoding, CC and NRMSE are 3.8% higher and 0.0096 lower and 6% higher, respectively. Statistical analysis shows that the proposed RoPE outperforms the Sinusoidal PE in terms of CC (*p* < 0.05).

#### 4.3.2 The effect of *μ*-law normalization

In the current research, we first study the performance of our RoFormer model with LSTM, TCN, Transformer, and CNN-Attention methods in subject-specific situations. We only utilized a single subject’s data to train the subject-specific models. Next, we study the performance of the cross-subject model, in which the training dataset is drawn from several subjects, and the test data is drawn from each individual in the training set. In the subsequent cross-subject experiments, the sEMG signals from all 10 subjects were combined and used as the training data.

To compare *μ*-law normalization and Z-score normalization, we apply them to subject-specific models and multi-agent models, respectively. In our study, *μ* is set to $2^{20}$ . The results depicted in Tables 1, 2 exhibit the substantial superiority of our method over other models, both in cross-subject and subject-specific cases. This illustrates the powerful ability of our proposed RoFormer method to extract features from the sEMG series. It is worth noting that models applying with *μ*-law regularization outperform models applying with Z-Score regularization in our study, especially in the cross-subject case. This is because *μ*-law normalization can better amplify low magnitudes and preserve the scale of larger values, while Z-Score normalization may disturb the original features of each subject itself when several subjects are analyzed together.

**Table 1:** Average performance of 6 movements on 10 subjects with *μ*-law normalization.

Model	Ave.PCC	Ave.NRMSE	Ave.R2
LSTM	0.6739	0.1383	0.4485
TCN	0.7288	0.1302	0.5107
Transformer	0.7719	0.1231	0.5682
CNN-Attention	0.8132	0.1120	0.6377
RoFormer	**0.8562**	**0.1090**	**0.6752**
LSTM*	0.5393	0.1750	0.1532
TCN*	0.6456	0.1651	0.2242
Transformer*	0.6935	0.1447	0.3901
CNN-Attention*	0.7694	0.1254	0.5374
RoFormer*	**0.8146**	**0.1205**	**0.5711**

* stands for the cross-subject model that was tested using each subject from the training set after being trained on all 10 subjects’ data. The value in bold is the best result.

**Table 2:** Average performance of 6 movements on 10 subjects with Z-score normalization.

Model	Ave.PCC	Ave.NRMSE	Ave.R2
LSTM	0.7963	0.1161	0.61484
TCN	0.8065	**0.1141**	0.6279
Transformer	0.7645	0.1246	0.5622
CNN-Attention	0.8157	0.1153	0.6389
RoFormer	**0.8176**	0.1149	**0.6402**
LSTM*	0.4812	**0.2026**	0.0614
TCN*	0.4349	0.2182	0.0329
Transformer*	0.4648	0.2048	0.0853
CNN-Attention*	0.4970	0.2162	0.0863
RoFormer*	**0.5345**	0.2047	**0.1173**

* stands for the cross-subject model that was tested using each subject from the training set after being trained on all 10 subjects’ data. The value in bold is the best result.

The RoFormer model performs similarly to the other models when Z-Score normalization is applied. However, when *μ*-law normalization is applied, significant improvements are brought to the RoFormer model, but the performance of other models is not greatly improved. Cross-subject models inevitably lead to performance degradation compared to subject-specific models, but this is acceptable.

#### 4.3.3 The effect of the ATL approach

When test subjects are not contained in the training set, the performance of the cross-subject model drops dramatically. Therefore, we propose an adversarial transfer learning (ATL) approach for improving the accuracy of finger joint angle estimation under cross-subject cases. During model calibration for each new individual, we first built the cross-subject model based on the training data from the other 9 subjects excluding the new individual, then calibrated the cross-subject network with the new user’s training data utilizing the ATL approach. Following a short calibration, the performance of five models was evaluated sequentially on 10 new users. Moreover, to highlight the improvement of the ATL approach on cross-subject model generalization performance, we compare it with the fine-tuning (FT) transfer learning method. In the following experiments, all models were applying *μ*-law normalization.

**Table 3:** Average performance of five models applying different transfer learning approaches.

Transfer learning approaches		Ave.PCC	Ave.NRMSE	Ave.R2
noTL	LSTM	0.420 ± 0.079	0.2113 ± 0.0113	0.202 ± 0.082
	TCN	0.423 ± 0.066	0.2065 ± 0.0102	0.227 ± 0.084
	Transformer	0.439 ± 0.059	0.2201 ± 0.0093	0.218 ± 0.076
	CNN-Attention	0.456 ± 0.054	0.2241 ± 0.0085	0.237 ± 0.071
	RoFormer	**0.458 ± 0.043**	**0.2047 ± 0.0076**	**0.260 ± 0.069**
FT	LSTM	0.526 ± 0.035	0.1601 ± 0.0086	0.270 ± 0.054
	TCN	0.631 ± 0.036	0.1503 ± 0.0087	0.344 ± 0.052
	Transformer	0.718 ± 0.035	0.1304 ± 0.0083	0.499 ± 0.048
	CNN-Attention	0.752 ± 0.033	0.1208 ± 0.0082	0.574 ± 0.045
	RoFormer	**0.776 ± 0.034**	**0.1205 ± 0.0081**	**0.601 ± 0.046**
ATL	LSTM	0.663 ± 0.029	0.1413 ± 0.0079	0.438 ± 0.046
	TCN	0.701 ± 0.030	0.1322 ± 0.0085	0.501 ± 0.045
	Transformer	0.766 ± 0.028	0.1264 ± 0.0074	0.552 ± 0.044
	CNN-Attention	0.800 ± 0.027	0.1184 ± 0.0073	0.617 ± 0.043
	RoFormer	**0.841 ± 0.026**	**0.1132 ± 0.0072**	**0.652 ± 0.043**

The value in bold is the best result.

We compared the average performance of five models applying different transfer learning approaches. NoTL indicates the cross-subject model that was tested using new individual data not in the training set without applying any transfer learning approach. The CC, NRMSE, and R2 were utilized as the performance metrics, respectively. As demonstrated in Figure 3, the FT strategy is applied to all models, where the average CC, NMRSE, and R2 of RoFormer are 0.776 ± 0.034, 0.1205 ± 0.0081 and 0.601 ± 0.046 respectively, which is better than the other four models. In terms of the performance of applying the ATL strategy, RoFormer also achieved the optimal performance average CC, NMRSE, and R2 were 0.841 ± 0.026, 0.1132 ± 0.0072, and 0.652 ± 0.043, respectively. Furthermore, two transfer learning strategies were applied to the Roformer model respectively, and compared with the FT approach, the CC of ATL was 6.5% higher (*p* < 0.05). Statistical analysis showed that the ATL approach was significantly better than the FT approach in terms of CC (*p* < 0.05), showing better generalization performance.

## 5 Discussion

To build a cross-subject model to estimate the hand kinematics of new subjects, this study proposes a RoFormer-based method with an ATL approach. First, the RoFormer network is utilized to extract features from several subjects when building a cross-subject model. Then, the parameters of the cross-subject network are updated by the ATL approach. On the NinaproDB2 dataset, the newly put forward cross-subject model’s performance is verified. Our cross-subject model offers better generalization performance in addition to improved accuracy for subject-specific finger kinematics estimation when compared with the other four subject-specific models and the FT approach. We also study the impact of different positional embedding, regularization methods, and transfer learning approaches on the model. This research contributes significantly to the generalization of deep learning approaches to robotic hand control and other HMI scenarios.

Firstly, in order to study the impact of RoPE on model performance, we compared the average performance of Transformer models applying different position embedding methods on 10 subjects of NinaproDB2. The results in Figure 5 showed that RoPE outperformed Sinusoidal position embedding and Learnable position embedding in terms of CC, NRMSE, and R2. This is because RoPE can capture the relative positional information of the sequence, thereby better representing the temporal relationships between multiple grasping actions. How to make use of the subject’s training data to enhance the generalization ability of the cross-subject model is a significant step. Among them, the *μ*-law normalization method plays a crucial role. Therefore, we compared the effects of different regularization methods on model performance in single-subject and cross-subject tasks. We train our proposed RoFormer model and LSTM, TCN, Transformer, and CNN-Attention models under different regularization methods, using CC, NRMSE, and R2 to measure the estimation quality. The results demonstrated that the *μ*-law normalization method greatly enhanced the cross-subject model’s performance. The proposed RoFormer model significantly outperforms other models both in single-subject and cross-subject cases, demonstrating that our method has a stronger generalization ability. The possible reason is that *μ*-law normalization can better exploit the hidden information of small-amplitude sEMG, while Z-Score normalization may disturb the original features of each subject itself when several subjects are analyzed together. Once each algorithm (LSTM, TCN, Transformer, CNN-Attention, and RoFormer) is applied to a cross-subject model, the performance declines substantially compared to the single-subject model. In particular, performance drops drastically when testing with a new individual that is not in the training set. Therefore, we propose an ATL approach to adapt the model to different subjects. The contribution of ATL to cross-subject modeling was further studied via comparison. The results in Figure 5 show that the ATL approach significantly improves the generalization ability across individual models in terms of CC, NRMSE, and R2 compared with the fine-tuning (FT) approach. FT only updates the parameters of the MLP layer within the cross-subject network. In contrast, ATL is designed to work adversarially, enabling domain transfer and utilizing source data to address target data insufficiency (Hung et al., 2018). Better parameter optimization may be the reason why ATL performs better than FT.

This study has a few limitations as well. Firstly, we deliberately avoid unreasonable or poor data caused by collection errors in NinaproDB2. In comparison to the subject adversarial knowledge (SAK) transfer learning strategy proposed by Long et al. (2023), we adopted the more efficient RoFormer model as the main body of the cross-subject model and utilized the *μ*-law normalization method to achieve more accurate estimation. Furthermore, we also simplify the structure of the domain discriminator. But there are also some shortcomings. The proposed method was only evaluated on six basic grasp actions performed by 10 intact subjects, which is far from covering the complex hand movements in our daily lives and also leads to insufficient robustness validation of our method. Secondly, the proposed RoFormer structure is based on attention mechanisms, which results in high inference latency. To accommodate the demands of real-world applications, the inference time should be cut down. Lastly, the newly proposed ATL approach often suffers from catastrophic forgetting (McCloskey and Cohen, 1989), compromising its ability to retain prior subject knowledge. In recent years, there have been some studies on lifelong learning methods (Hadsell et al., 2020) that can effectively address catastrophic forgetting, which can serve as a direction for our future research and improvement.

## 6 Conclusion

In the present study, we proposed a RoFormer-based cross-subject model for continuously estimating finger kinematics and an adversarial transfer learning(ATL) approach to improve the cross-subject model’s generalization ability. RoFormer-based model can extract spatial and temporal information from sEMG, allowing them to better satisfy the needs of cross-subject scenarios of clinical settings. Simultaneously, *μ*-law normalization is adopted to replace Z-Score normalization in order to better exploit the hidden information of small-amplitude sEMG signals. Subsequently, four classic models for continuous motion estimation and a fine-tuning-based transfer learning approach are compared with our RoFormer-based cross-subject model and ATL approach and validated on NinaproDB2. Future studies can incorporate more subjects, actions, and channels to assess the robustness and stability of the model. Recently, there have been many studies on efficient Transformers (Tay et al., 2020), among which Performers based on linear attention can improve model efficiency and shorten model inference time. RoPE is the sole kind of relative position embedding that can currently be utilized for linear attention and can be used as a direction for future follow-up. Catastrophic forgetting refers to the drastic decline in the performance of a model on the original task after being trained on a new task. Transfer learning strategies often suffer from catastrophic forgetting. Lifelong learning can effectively improve the catastrophic forgetting problem and adapt the model to new subjects while retaining the knowledge of old subjects. It is anticipated that research on lifelong learning approaches or transfer learning strategies based on the Transformer model will make more contributions to human-computer interaction.

## Data availability statement

The original contributions presented in the study are included in the article/supplementary material, further inquiries can be directed to the corresponding author.

## Author contributions

CL: Conceptualization, Investigation, Software, Supervision, Writing – original draft. ZH: Data curation, Formal analysis, Methodology, Validation, Visualization, Writing – original draft, Writing – review & editing.
